# CBCT Assessment of Mental Foramen Position Relative to Anatomical Landmarks

**DOI:** 10.1155/2016/5821048

**Published:** 2016-11-23

**Authors:** Mahnaz Sheikhi, Mitra Karbasi Kheir

**Affiliations:** ^1^Torabinejad Research Center, Department of Oral and Maxillofacial Radiology, School of Dentistry, Isfahan University of Medical Sciences, Isfahan, Iran; ^2^Department of Oral and Maxillofacial Radiology, School of Dentistry, Islamic Azad University Isfahan, Khorasgan Branch, Isfahan, Iran

## Abstract

*Purpose*. This study was carried out on an Iranian population aiming to investigate mental foramen position relative to inferior border of mandible and skeletal midline and its gender and age differences on CBCT projections.* Materials and Methods*. A number of 180 CBCT images of patients were analyzed in different planes (tangential, cross-sectional, and axial). The distances from the superior border of mental foramen to the inferior border of mandible and from the anterior border of mental foramen to the midline were calculated.* Results*. The mean distance from mental foramen to the inferior border of mandible in the right side was 13.26 mm (SD ± 2.34) and in the left side was 13.37 mm (SD ± 2.19). There was a statistically significant difference between genders in terms of the distance between mental foramen and inferior border of mandible (*P* value = 0.000). The mean distances from mental foramen to midline were 25.86 mm (SD ± 0.27) and 25.53 mm (SD ± 0.31) in the right and left sides, respectively.* Conclusions*. The vertical and horizontal positions of mental foramen can be determined from stable anatomical landmarks such as mandibular inferior border and skeletal midline in both dentulous and edentulous patients. The distance from the superior border of mental foramen to the inferior border of mandible exhibited sexual dimorphism.

## 1. Introduction

Mental foramen is the opening of mandibular canal in the body of mandible, and mental foramen position shows variations. As mental foramen is a critical landmark in implant surgery, determining the mental foramen position can help to gain sufficient local anesthesia and to decrease iatrogenic injuries during surgical treatments. Some studies have determined the vertical and horizontal positions of mental foramen according to the adjacent premolars and molars on the skull, conventional radiographs, and CBCT [1–7]. Nevertheless, many patients who need treatments in this region have partially or completely lost their teeth; so other stable anatomical landmarks are superior to be used to locate the foramen position. There are a few studies, mostly on skulls, which have measured the distance of mental foramen to the anatomical landmarks. The mandibular inferior border and skeletal midline landmarks have been used to locate the horizontal and vertical positions of mental foramen in these studies [8–10]. However, these measurements have not been done on cone-beam computed tomography (CBCT). This study was conducted on an Iranian population to determine mental foramen positions relative to inferior border of mandible and skeletal midline and its age and gender differences on CBCT projections.

## 2. Materials and Methods

A total number of 180 CBCT images, taken from the patients for diagnostic purposes in the department of maxillofacial radiology at Isfahan school of dentistry from 2010 to 2015, were examined by two examiners (two maxillofacial radiologists) using Sirona GALILEOS software on LG LED computer viewer (E2042C, Korea). All CBCT images were taken by Sirona Orthophos, GALILEOS version 1.7 (Sirona, Germany), and flat panel detector. The adjusted scan parameters were 85 Kvp and 10–42 mA depending on the size of the patients. The exposure time was 14 seconds, effective exposure time was 2–6 seconds, and voxel size was 0.3*∗*0.3*∗*0.3 mm. The inclusion criteria comprised the patients older than 18 years whose skeletal growth had been completed (partial or full edentulous and dentate patients) and the exclusion criteria included the patients with pathologic lesions in mandible.

**Figure 1:**
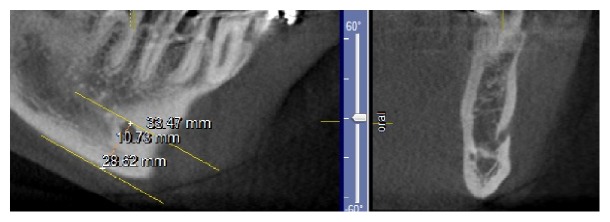
The distance from the superior border of mental foramen to the inferior border of mandible.

CBCT projections were analyzed in different planes (tangential, cross-sectional, and axial). Mental foramen was identified in cross-sectional and axial views. To determine mental foramen position relative to the inferior border of mandible, two parallel lines were drawn in the tangential section, from the upper border of mental foramen and inferior border of mandible, using length measuring option on GALILEOS software. The distance between these two lines was measured by drawing a perpendicular line on them (Figure 1). To measure the distance of foramen to midline, two parallel lines were drawn in the tangential section, from the anterior border of the foramen and midline, using length measuring option on GALILEOS software. Because the dentate and edentulous patients were examined, the skeletal midline was chosen, and mandibular lingual foramen was chosen as the indicator to draw the midline. The finding option was selected and eight cross-sectional images with the least slice thickness, 0.3 mm, were produced and each line was placed in a separate slice. The distance between these two lines was calculated by the distance between the slices.

Comparisons were made between genders, sides, and mean ages. The data were analyzed by the statistical package for social sciences (SPSS) (version 22, SPSS Inc., Chicago, IL).

## 3. Results

From 180 CBCT images, 84 and 96 images belonged to men and women, respectively. The mean age of the patients was 48.6 years. There were 75% partial edentulous, 17.2% full edentulous, and 7.8% dentate cases in the right side and 75% partial edentulous, 16.7% full edentulous, and 8.3% dentate cases in the left side. Mental foramen was present in both sides in all of the images and the mean diameter of mental foramen was 3.59 mm (SD ± 2.74) in the right side and 3.59 mm (SD ± 1.17) in the left side.

The mean distance from the superior border of mental foramen to the inferior border of mandible (MF-MB) in the right side was 13.26 mm (SD ± 2.34) and in the left side was 13.37 mm (SD ± 2.19). The results of paired *t*-test showed no statistically significant differences in MF-MB distance between the right and left sides (*P* value = 0.488). There were statistically significant differences between genders in MF-MB (*P* value = 0.000), and MF-MB distances were greater in men than in women (Table 1). Moreover, there were no statistically significant differences between the mean age and MF-MB distance (right side: *R* = −0.105, *P* value = 0.162; left side: *R* = −0.04, *P* value = 0.598).

The mean distance from the anterior border of mental foramen to midline (MF-MM) was 25.86 mm (SD ± 0.27) in the right side and 25.53 mm (SD ± 0.31) in the left side. The result of paired sample *t*-test showed no statistically significant differences between the right and left sides in MF-MM distance (*P* value = 0.112). There was no statistically significant difference between genders in MF-MM (right *P* value = 0.90; left *P* value = 0.58) (Table 1). Also, there were no statistically significant differences between the mean age and MF-MM distance (right side: *R* = −0.131, *P* value = 0.08; left side: *R* = −0.122, *P* value = 0.104).

## 4. Discussion

Mental foramen is one of the most important landmarks of mandible for local anesthesia administration, surgical procedures, and forensic dentistry. It transmits the mental nerves and vessels that supply the chin, lower lip, buccal mucosa of incisors, canines, and premolars. Surgical trauma will cause paresthesia of the lip, chin, and oral mucosa that is often associated with a limited xerostomia [11, 12].

The mean distance from mental foramen to the inferior border of mandible, in our study, was in accordance with the results of the studies by Agthong et al. and Neiva et al. that were performed on skulls. They reported the mean ranges of 14-15 mm and 10.33–13.67 mm, respectively [8, 9]. Our mean range was slightly wider than those studies. Iranian population is mostly Caucasian, but as this study showed, it had a wider range than those reported by Neiva et al. This means that in Iranian population mental foramen is closer to alveolar crest compared to other Caucasians. Consequently, with alveolar bone resorption after tooth extraction, mental foramen gets closer to alveolar crest and less bone height will remain for implant drilling. As literature has shown, with tooth loss and alveolar bone resorption, the mental foramen moves upwards closer to the alveolar border and in severe resorption, the mental nerve and the final part of the inferior alveolar nerve may be found at the alveolar margin or even under the gums [13, 14]. Apinhasmit et al. reported the mean distance between mental foramen and bottom of mandible to be 14.33 mm in direct measurement and 16.52 mm in panoramic assessment [10]. Our results were more similar to the results of direct measurement of the above study; so it can show that the results of measurement on CBCT are closer to those of direct measurement.

This study revealed a statistically significant difference between gender and the distance from superior border of mental foramen to the inferior border of mandible, indicating a higher distance in males than in females. We could not find similar studies on CBCT, but there are some studies on panoramic radiographs. Our results were in agreement with those of Chandra et al. who studied panoramic radiographs of a population in North India. They concluded that the distances from the superior and inferior borders of mental foramen to inferior border of mandible exhibited sexual dimorphism, showing higher distances in males than in females [15]. Likewise, the results were in agreement with those of Thomas et al., Catovie et al., Mahima et al., and Thakur et al. [16–19]. Although most of the studies have been done on panoramic radiographs, studies have shown the results of CT scans are more accurate than conventional radiographs to detect mental foramen [11], so CBCT studies are superior to panoramic ones. The similar results in sex predilection of mental foramen distance to inferior border of mandible may be considered as an aid in sex determination. However, because of the overlap between the ranges in Iranian males and females, it may not be used on an individual.

The findings of this study were in accordance with the results of Lim et al.' study which found mental foramen position changed during primary dentition and remained mostly stable during the eruption of the primary and mixed dentitions [20]. Our samples were above 18 years of age; therefore, the distance from mental foramen to midline and inferior border of mandible was constant between different ages. On an assessment of panoramic radiographs, literature has found that the distance from the foramen to the inferior border of mandible remains relatively constant throughout life despite the resorption of alveolar process above the foramen, which was confirmed by our CBCT results [21–23]. In the study of Ngeow et al. performed on panoramic radiographs, nonvisibility of the foramen was greatly increased in patients aged ≥ 50 years because of slower bone remodeling [24]. In the present study carried out on CBCT projection, mental foramen was visible in all patients in both sides. Unlike panoramic radiograph, CBCT can display mental foramen in aged patients despite bone porosity and slower bone remodeling.

The distance from the anterior border of mental foramen to the midline was not in line with the results of the studies of Agthong et al., Neiva et al., and Apinhasmit et al. that were performed on skulls. They reported range of 28 mm, 27.61 mm, and 28.52 mm, respectively [8, 9]. In our study, the distance was less than those reported by the above studies, indicating a shorter interforaminal distance (safe zone) in Iranian population.

## 5. Conclusions

On a CBCT assessment in an Iranian population, it is possible to conclude that the vertical and horizontal positions of mental foramen can be determined from stable anatomical landmarks such as mandibular inferior border and skeletal midline in both dentulous and edentulous patients. The mean distance from the superior border of mental foramen to the inferior border of mandible exhibited sexual dimorphism but there was no sex predilection in the distance from the anterior border of mental foramen to midline.

## Figures and Tables

**Table 1:** The distance from superior border of mental foramen to inferior border of mandible (distance MF-MB) and the distance from anterior border of mental foramen to mandibular midline (distance MF-MM).

Variables	Gender	Number	Mean distance MF-MB	Mean distance MF-MM
Right side	Male	84	14.36 mm (SD ± 2.07)	25.82 mm (SD ± 3.87)
	Female	96	12.30 mm (SD ± 2.13)	25.89 mm (SD ± 3.64)
Left side	Male	84	14.38 mm (SD ± 2.01)	25.35 mm (SD ± 4.77)
	Female	96	12.48 mm (SD ± 1.95)	25.69 mm (SD ± 3.57)
